# IRGM1 supports host defense against intracellular bacteria through suppression of type I interferon in mice

**DOI:** 10.1172/JCI171982

**Published:** 2023-11-01

**Authors:** Prashant Rai, Martin Sharpe, Charan K. Ganta, Paul J. Baker, Katrin D. Mayer-Barber, Brian E. Fee, Gregory A. Taylor, Michael B. Fessler

**Affiliations:** 1 Immunity, Inflammation and Disease Laboratory and; 2 Comparative & Molecular Pathogenesis Branch, National Institute of Environmental Health Sciences, NIH, Research Triangle Park, North Carolina, USA.; 3 Laboratory of Clinical Immunology and Microbiology, National Institute of Allergy and Infectious Diseases, NIH, Bethesda, Maryland, USA.; 4 Department of Medicine and Center for the Study of Aging and Human Development,; 5 Department of Molecular Genetics and Microbiology, and; 6 Department of Immunology, Duke University Medical Center, Durham, North Carolina. USA.; 7 Geriatric Research Education and Clinical Center, Durham VA Health Care System, Durham, North Carolina, USA.

**Keywords:** Immunology, Infectious disease, Bacterial infections, Innate immunity, Macrophages

**To the Editor:** IFN-γ enhances cell-autonomous host defense by inducing several families of antimicrobial target genes, including immunity-related GTPases (IRGs). Animals deficient in IRGM1, the best-studied IRG, succumb to numerous bacterial and protozoal infections in a manner that nearly phenocopies that of IFN-γ–null mice (1). This infection susceptibility has been attributed to the cell-intrinsic role of IRGM1 in xenophagy and targeting of pathogen-containing vacuoles (1).

Recently, we reported that *Irgm1^-/-^* mice spontaneously produce excess type I IFN (IFN-I) (2). Although IFN-I is protective against virus, it can compromise antibacterial host defense (3). We hypothesized that IFN-I, rather than defective cell-intrinsic defenses, drives the susceptibility of *Irgm1^–/–^* mice to bacteria. Consistent with this, we found that *Irgm1^–/–^* mice succumbed to *Mycobacterium tuberculosis* and *Listeria monocytogenes*, as previously reported (1), but *Irgm1^–/–^* mice lacking the IFN-I receptor, IFNAR1 (i.e., *Irgm1^–/–^Ifnar^–/–^* mice), were resistant (Figure 1A). Similarly, the increased pathogen burden in *Irgm1^–/–^* mice following infection with *Salmonella typhimurium* was normalized in *Irgm1^–/–^Ifnar^–/–^* mice (Figure 1A). By contrast, during infection with *Toxoplasma gondii*, a pathogen for which IFN-I is host protective (4), *Irgm1^–/–^* mice had reduced survival, and this was not rescued in *Irgm1^–/–^Ifnar^–/–^* animals (Supplemental Figure 1A; supplemental material, including the Supplemental Methods, available online with this article; https://doi.org/10.1172/JCI171982DS1).

To investigate IFN-I’s mechanism of compromising host defense in *Irgm1^–/–^* mice, we pursued the *L*. *monocytogenes* infection model. After infection, *Irgm1^–/–^* mice had elevated biomarkers of organ damage in sera (Supplemental Figure 1B) and increased inflammation and necrosis in livers and spleens (Supplemental Figure 1, C–E), phenotypes that were rescued in *Irgm1^–/–^Ifnar^–/–^* mice. Increased cell death in *Irgm1^–/–^* livers and spleens was dependent on IFN-I signaling (Figure 1, B and C, and Supplemental Figure 1, F and G). Compared with that in WT and *Irgm1^–/–^Ifnar^–/–^* organs, there was increased *L*. *monocytogenes* growth in *Irgm1^–/–^* organs (Supplemental Figure 1, H–J). Increased growth was seen by 4 hours after infection in the peritoneum (Figure 1D), the site of *L*. *monocytogenes* inoculation in our model, indicating that IFN-I suppresses clearance of *L*. *monocytogenes* upon initial encounter. Indeed, *Irgm1^–/–^* F4/80^hi^ peritoneal macrophages internalized *L*. *monocytogenes* normally in vitro (Supplemental Figure 2A) but had reduced killing capacity (Figure 1E). This was associated with decreased lysosomal delivery of *L*. *monocytogenes* (Figure 1F and Supplemental Figure 2B), despite normal lysosomal mass (Supplemental Figure 2C) and pH (data not shown) in *Irgm1^–/–^* macrophages. *L*. *monocytogenes*–challenged *Irgm1^–/–^* F4/80^hi^ peritoneal macrophages also had higher expression levels of STAT1, STAT2, (Y701-)PO_4_-STAT1, and (Y689-)PO_4_-STAT2 than their WT and *Irgm1^–/–^Ifnar^–/–^* counterparts (data not shown). In vivo, 4 hours after infection by GFP-expressing *L*. *monocytogenes*, only *Irgm1^–/–^* F4/80^hi^ macrophages showed increased bacterial load (Figure 1G and Supplemental Figure 2D).

Given that IFN-I may induce cell death (3), we examined peritoneal myeloid cells for viability (Supplemental Figure 2E). Lytic death was increased in the *Irgm1^–/–^* peritoneum on days 1 and 3 after infection and was IFN-I–dependent (Supplemental Figure 3A). Fewer neutrophils were recruited by 4 hours after *L*. *monocytogenes* infection to *Irgm1^–/–^* peritonea, but neutrophil accumulation increased dramatically after 24 hours in an IFN-I–dependent manner (Figure 1H), and increased citrullinated histones, a marker of lytic neutrophil death by NETosis, were detected on day 3 (Supplemental Figure 3B). Increased IFN-I–dependent lytic death was also observed among F4/80^hi^ macrophages (Supplemental Figure 3A), perhaps explaining their depletion 24 hours after infection (Figure 1, I and J). Notably, increased staining of phosphorylated mixed lineage kinase domain–like pseudokinase, a necroptosis effector, was observed only in *Irgm1^–/–^* macrophages (Supplemental Figure 3, C and D). Thus, IFN-I promotes multiple modes of proinflammatory lytic cell death in *Irgm1^–/–^* mice. Accordingly, *Irgm1^–/–^* peritoneal fluid exhibited an IFN-I–dependent increase in lactate dehydrogenase activity and proinflammatory cytokines (Supplemental Figure 3, E and F).

During peritonitis, death of resident macrophages leads to recruitment and reprogramming of Ly6C^hi^F4/80^–^ monocytes into Ly6C^–^ F4/80^hi^ macrophages, often through an MHCII^+^F4/80^lo/int^ intermediate (5). We observed emergence of a small F4/80^+^ population on day 3 after *L*. *monocytogenes* infection (Supplemental Figure 2E). Unlike their WT, *Ifnar^–/–^*, and *Irgm1^–/–^Ifnar^–/–^* counterparts, all CD11b^+^F4/80^hi^ macrophages in the *Irgm1^–/–^* peritoneum at day 3 after *L*. *monocytogenes* infection retained high Ly6C and did not express TIM4 (Supplemental Figure 4, A and B), a maturity marker of peritoneal macrophages (5). The CD11b^+^F4/80^lo^ population in *Irgm1^–/–^* animals remained Ly6C^hi^ at day 3 and lacked a MHCII^+^ subpopulation (Supplemental Figure 4C). The receptor for colony-stimulating factor-1 (CD115), which is critical for survival and differentiation of monocytes, was repressed in *Irgm1^–/–^* Ly6C^hi^ cells in an IFN-I–dependent manner (Supplemental Figure 4C). Ly6C^hi^ monocytes were also elevated in *Irgm1^–/–^* blood and showed reduced CD115 and MHCII (Supplemental Figure 4E). These results suggest that excess IFN-I in *Irgm1^–/–^* mice impairs maturation of inflammatory Ly6C^hi^ monocytes into macrophages, possibly by repressing CD115.

To specifically examine myeloid IFN-I signaling, we infected *Irgm1^–/–^* mice lacking IFNAR1 solely in myeloid cells (*Irgm1^–/–^LysM*:Cre^+^*Ifnar*^Fx/Fx^ mice). These mice showed decreased necrotic death of peritoneal myeloid cells, partial rescue of CD115 in CD11b^+^F4/80^lo^Ly6C*^-^* cells (Supplemental Figure 4, F and G), and reduced bacterial burden (Figure 1K) compared with controls, indicating that myeloid IFN-I signaling compromises myeloid cell fate and host defense in *Irgm1^–/–^* mice.

Our findings challenge the long-prevailing paradigm that IRGM1 serves as an IFN-γ–induced cell-autonomous host defense effector (1) and suggest instead that IRGM1 supports host defense by preventing excess autocrine and/or paracrine IFN-I from compromising myeloid cell fate and function. Future studies will be required to distinguish autocrine versus paracrine mechanisms.

## Supplementary Material

Supplemental data

Supporting data values

## Figures and Tables

**Figure 1:**
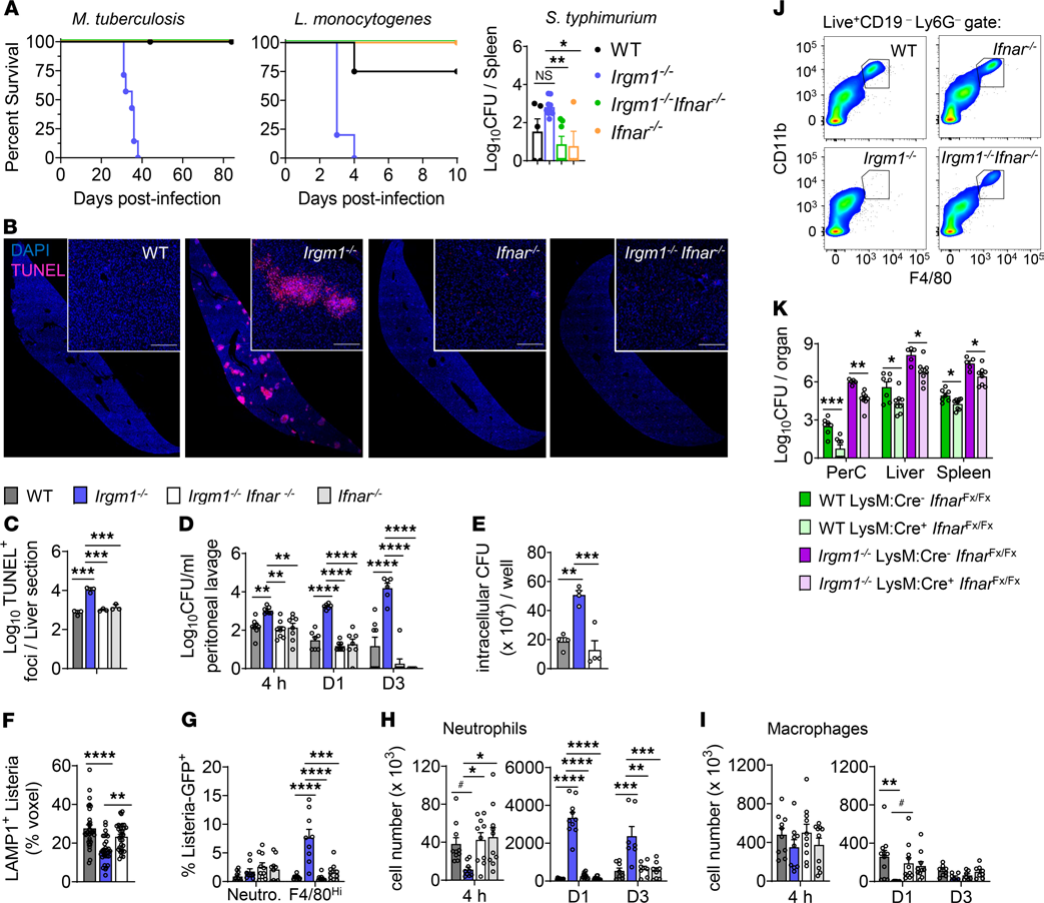
IFN-I induces susceptibility to bacterial infection in *Irgm1*^–/–^ mice. (**A**) Survival curves after infection with *M*. *tuberculosis* (*n* = 7–10) and *L*. *monocytogenes* (*n* = 4–5). Spleen CFU on day 21 after *S*. *typhimurium* infection (*n* = 4–9). (**B**) Liver on day 3 after *L*. *monocytogenes* infection stained for TUNEL and DAPI. Scale bar: 200 μm. (**C**) Quantification of TUNEL^+^ foci (*n* = 3). (**D**) Peritoneal lavage CFU at 4 hours, day 1, and day 3 after *L*. *monocytogenes* (*n* = 4–8). (**E**) Isolated F4/80^hi^ peritoneal macrophages exposed to *L*. *monocytogenes* were permeabilized for CFU count after 24 hours. (**F**) Macrophages were stained for *L*. *monocytogenes* and lysosome (LAMP1) at 6 hours and quantified for volumetric pixels of *L*. *monocytogenes* that were LAMP1^+^ (*n* = 32 images). (**G**) Percentage GFP^+^ after 4-hour infection by GFP-tagged *L*. *monocytogenes* (*n* = 9–10). (**H**) Neutrophil and (**I**) F4/80^hi^ tissue macrophage numbers in infected peritoneal lavage (*n* = 7–11). (**J**) Representative plot showing depletion of CD11b^+^F4/80^hi^ macrophages at 24 hours. (**K**) CFU in peritoneal cavity (PerC), liver, and spleen on day 3 after *L*. *monocytogenes* (*n* = 5–8). Data are shown as the mean ± SEM. ^#^*P* < 0.08, *P < 0.05, **P < 0.01, ***P < 0.001, and ****P < 0.0001 (1-way ANOVA with Tukey’s adjustment for **A** and **C**–**I** or Student’s *t* test for **K**).

## Version 1 09/12/2023

In-Press Preview

## Version 2 11/01/2023

Electronic publication
