# Supplementary material for: IRGM1 supports host defense against intracellular bacteria through suppression of type I interferon in mice
Source: J Clin Invest. 2023 Nov 1;133(21):e171982. doi: 10.1172/JCI171982 (PMC10617763; doi:10.1172/JCI171982)
Supplement: Supplemental data [file jci-133-171982-s025.pdf]

IRGM1 supports host defense against intracellular bacteria through suppression of type I interferon in mice

Prashant Rai[1], Martin Sharpe[1], Charan K. Ganta[2], Paul J. Baker[3], Katrin D. Mayer-Barber[3], Brian E. Fee[4], Gregory A. Taylor[4,5,6,7], Michael B. Fessler[1]

[1]Immunity, Inflammation and Disease Laboratory and [2]Comparative & Molecular Pathogenesis Branch, National Institute of Environmental Health Sciences, NIH, Research Triangle Park, NC
[3]Inflammation and Innate Immunity Unit, Laboratory of Clinical Immunology and Microbiology, National Institute of Allergy and Infectious Diseases, NIH, Bethesda, MD
[4]Department of Medicine, Division of Geriatrics, and Center for the Study of Aging and Human Development;
[5]Department of Molecular Genetics and Microbiology; and
[6]Department of Immunology, Duke University Medical Center, Durham, NC 27710
[7]Geriatric Research, Education, and Clinical Center, Durham VA Health Care System, Durham, NC 27705

Supplemental Material

Methods

Animals

Irgm1$^{-/-}$ mice have been described before (1) and were backcrossed more than eight generations onto the C57BL/6 background. *Ifnar*$^{-/-}$ (stock no. 032045, Jackson Laboratory) mice were eight generations backcrossed to C57BL/6 before crossing with *Irgm1*$^{-/-}$ mice. Wild-type controls were either littermates or mice from the same breeding colony.

Mycobacteria infection

Animals placed in a whole-body inhalation system (Glas-Col; Terre Haute, IN) were exposed to aerosolized *Mycobacterium tuberculosis* H37Rv strain (100–200 CFU/mouse). Delivery doses were determined by measuring lung CFU 2–24 h postexposure from three to five control mice (2).

Salmonella infection

Salmonella typhimurium aroA- strain SL7731 was cultured overnight in LB broth without shaking. Mice were inoculated by oral gavage with 0.2 mL PBS containing 1 x 10^9 CFU. 21 days later, organs were isolated aseptically, homogenized in sterile PBS, serially diluted, and plated on LB plates. CFUs were counted 24h later.

Toxoplasma infection

Toxoplasma gondii type II A7 Prugniaud strain tachyzoites were propagated in Hs27 human foreskin fibroblasts in DMEM supplemented with 10% FBS, penicillin (100U/ml), and streptomycin (100 µg/ml). Mice were infected intraperitoneally with 200 tachyzoites in 100 µl sterile PBS and monitored for survival with a 20% weight loss end point.

Listeria infection

Listeria monocytogenes 10403S strain (LM) were grown in brain-heart infusion (BHI) broth overnight at 37°C, resuspended in sterile PBS and diluted appropriately to inject ~1 x 10^4 CFU in 100 µl PBS intra-peritoneally. Mice were monitored for survival with a 20% weight loss end point. GFP-expressing *L. monocytogenes* 10403S strain was a kind gift from Dr. Darren E. Higgins (3).

For in vitro studies, bacteria were diluted in DMEM/10% FBS without antibiotics and infected at MOI of 7-12 for one hour. Cells were then washed twice gently with warm PBS and incubated in media containing 50 µg/ml gentamicin (Sigma) for required time.

Bacteria killing assay

F4/80^{Hi} peritoneal macrophages (0.3 x 10^6) seeded in 24-well plates were infected with LM in 200 µl media, as described above. After 1h and 24h, cells were washed once with PBS and permeabilized with sterile 0.5% Triton X-100 for 2 min, mixed by pipetting, serially diluted, and plated on BHI agar plates.

Peritoneal lavage measurements

Peritoneum was lavaged using 10 ml sterile PBS. Lavages were spun down, peritoneal cells were used for flow cytometry or macrophage isolation and supernatants were stored at -80°C. For analysis of peritoneal fluid, supernatants were thawed and concentrated 7.5 to 8 times using Amicon Ultra 0.5 ml centrifugal unit (Millipore UFC501024). For cytokine analysis, concentrated lavages were diluted twice, and cytokines were quantified by multiplex assay (Bio-Plex; Bio-Rad Laboratories). Final cytokine concentrations were adjusted by respective concentration or dilution factor. For citrullinated histone H3 (Cayman #501620), similar concentrated lavages

were used. To determine LDH activity (Sigma), neat (without concentration) peritoneal lavages were used.

Peritoneal macrophage isolation
Peritoneal cells were resuspended at 1 x 10^8 cells/ml in PBS with 1mM EDTA and 1% FBS. Cells were stained with 25 μg/ml anti-F4/80-biotin antibody (Biolegend, #123105) and Fc block (Biolegend #101302) for 30 min on ice, and magnetically sorted with EasySep™ Mouse Biotin Positive Selection Kit II (Stemcell). Peritoneal macrophages were grown in DMEM with 10% FBS, Penicillin/Streptomycin, 0.5 μM all-trans retinoic acid (ATRA) (Biogems #3027949) and 20 ng/ml M-CSF (Biolegend #576406), as previously described (4).

Flow Cytometry
1 x 10^6 peritoneal cells were stained with antibody cocktail in PBS containing 0.5% BSA, 2 mM EDTA, mouse and rat serum (5% each) and Fc block (Biolegend). Antibodies against following surface markers were used from Biolegend: CD115-BV605 (#135517), CD226-BV650 (#133621), TCRb-BV650 (#109251), F4/80-BV711 (#123147), CD19-FITC (#115506), CD19-PE-Cy7 (#115520), CD19-PerCPCy5.5 (#152406), Ly6C-PerCPCy5.5 (#128012), Ly6C-AF488 (#128022), Ly6G-FITC #(127606), Ly6G-PE (#127608), TIM4-PE-Cy7 (#130010), Ly6C-PE-Cy7 (#128018), CD19-PE-Cy7 (#115520), Ly6C-AF647 (#128016) and Annexin V-APC (#640920). Fixable Viability Dye eFluor™ 780 (# 65-0865-14), MHCII-eF450 (#48-5320-82) and MHCII-PE-Cy7 (#25-5321-82) were from eBioscience. CD11b-BUV395 was from BD Bioscience (#563553). After surface staining, cells were washed in PBS and stained with Annexin V in Annexin V binding buffer (R&D). Samples were fixed with BD Cytofix (BD #554655) before analyzing on flow cytometry (BD LSR Fortessa instrument; BD FACSDiva and FlowJo software for analysis).

Lysosome staining
1 x 10^6 peritoneal cells were stained with LysoTracker Deep Red (Invitrogen no. L12492) at 50 nM in 250 ul complete DMEM media for 30 min at 37°C, washed and surface stained as mentioned above for Flow cytometry.

TUNEL assay
Tissue sections were deparaffinized and treated with Proteinase K for 30 min at 37°C, washed with PBS, incubated with 100 μl TUNEL labeling enzyme (Roche #11684795910) for 1h at 37°C, washed with PBS again and mounted for microscopy.

Bacteria count in tissues
Spleen and liver were homogenized in 0.1% Triton X-100 PBS using OMNI TH hand-held homogenizer. Fresh peritoneal lavage in PBS was treated with Triton X-100 to a concentration of 0.1%. Bacteria were plated on BHI agar after appropriate serial dilution.

Confocal Microscopy
Isolated F4/80^{Hi} peritoneal macrophages (0.3 x 10^6) seeded on glass-bottom dish (MatTek) were infected with LM (MOI ~7 in 200 μl media) for an hour, washed and further incubated for 5 hours. Peritoneal lavage cells (~0.3 x 10^6) were cytospun on to glass slides and allowed to air dry overnight. Cells were fixed using 4% paraformaldehyde, permeabilized with 0.1% Triton X-100 and blocked with 3% BSA solution. Macrophages were stained for Listeria (1:2000, #ab35132) and LAMP1 (1:50, DSHB #1D4B) for 1h at room temperature followed by secondary antibody (Alexa Fluors, Invitrogen). Peritoneal lavage cells were stained for F4/80 (1:100, #ab6640) and phospho-MLKL (Ser345) (1:100, Millipore MABC1158). Cells were mounted with a cover slip (1.5 thickness, 22 × 50-mm or 12-mm diameter) on ProLong Diamond Antifade

Mountant with DAPI (Invitrogen). Images were taken in a Zeiss LSM 780 inverted confocal microscope with ×63 1.4 numerical aperture oil-immersion objective.

For F4/80^{Hi} macrophages, 8 slices were taken in the z plane. Surfaces of Listeria and lysosomes were constructed using Imaris software. To determine the voxels of Listeria that colocalized with lysosomes, lysosomal channel was masked by setting the inside voxel to 1. The intensity sum of the new masked channel was then divided by the total voxels of Listeria to determine percentage of Listeria localizing with the lysosome. For peritoneal lavage cells, the total intensity of phospho-MLKL only in F4/80-positive cells was determined using Imaris. For TUNEL-stained tissue sections, ImageJ was used to determine numbers of TUNEL-positive foci.

Histopathology and immunohistochemical analysis
Harvested tissues were fixed in 10% neutral buffered formalin, trimmed, processed for paraffin embedding, sectioned (5 μm), and stained with hematoxylin and eosin. The slides were scanned using an Aperio slide scanner (Leica Biosystems) and images were captured using Aperio's ImageScope. Tissues were evaluated for pathology by a board-certified veterinary pathologist. Immunohistochemical staining for Listeria using the Rb DAB Xtra Wash IHC protocol on the Leica Bond stainer, NR, anti-Listeria (BD, Cat# 223021, Lot# 2069070, assumed 1.0 mg/ml) or normal rabbit IgG at 1:5000, Bond Refine HRP detection and DAB visualization was done.

Enzyme activity in serum
ALT, AST, LDH, and CK reagents were purchased from Beckman Coulter, Inc. All enzymes were assayed using the AU480 clinical analyzer (Beckman Coulter, Inc).

Statistical analysis
Analysis was performed using GraphPad Prism software. Data are represented as mean ± SEM. One-way ANOVA and two-tailed Student's t test were used. For all tests, $P<0.05$ was considered significant. In Figure 1A, a single datapoint in *Irgm1*$^{-/-}$*Ifnar*$^{-/-}$ group (*S. typhimurium*) was found to be an outlier by ROUT (Q=1%) test and was excluded from analysis.

Study approval
Animals were used in accordance with the Animal Welfare Act and the US Public Health Service Policy on Humane Care and Use of Laboratory Animals. Experiments were reviewed by the Animal Care and Use Committee of the National Institute of Environmental Health Sciences (NIEHS) and the National Institute of Allergy and Infectious Diseases (NIAID).

Data availability
Data are available in the 'Supporting data' document online and also from the authors upon reasonable request.

Acknowledgements

We thank L. Perrow for assistance with breeding; NIEHS Pathology Core laboratory for assistance with processing, sectioning, and staining of tissues; C.J. Tucker and E. Scappini of the NIEHS Fluorescence Microscopy and Imaging Center; and C. Bortner and M. Sifre of the NIEHS Flow Cytometry Core Facility. This research was supported by the Intramural Research Program of the NIH, National Institute of Environmental Health Sciences.

References

1. Collazo CM, Yap GS, Sempowski GD, Lusby KC, Tessarollo L, Vande Woude GF, et al. Inactivation of LRG-47 and IRG-47 reveals a family of interferon gamma-inducible genes with essential, pathogen-specific roles in resistance to infection. *J Exp Med.* 2001;194(2):181-8.
2. Bohrer AC, Tocheny C, Assmann M, Ganusov VV, and Mayer-Barber KD. Cutting Edge: IL-1R1 Mediates Host Resistance to Mycobacterium tuberculosis by Trans-Protection of Infected Cells. *J Immunol.* 2018;201(6):1645-50.
3. Tan JMJ, Garner ME, Regeimbal JM, Greene CJ, Marquez JDR, Ammendolia DA, et al. Listeria exploits IFITM3 to suppress antibacterial activity in phagocytes. *Nat Commun.* 2021;12(1):4999.
4. Davies LC, Rice CM, Palmieri EM, Taylor PR, Kuhns DB, and McVicar DW. Peritoneal tissue-resident macrophages are metabolically poised to engage microbes using tissue-niche fuels. *Nat Commun.* 2017;8(1):2074.

Supplemental Figure 1

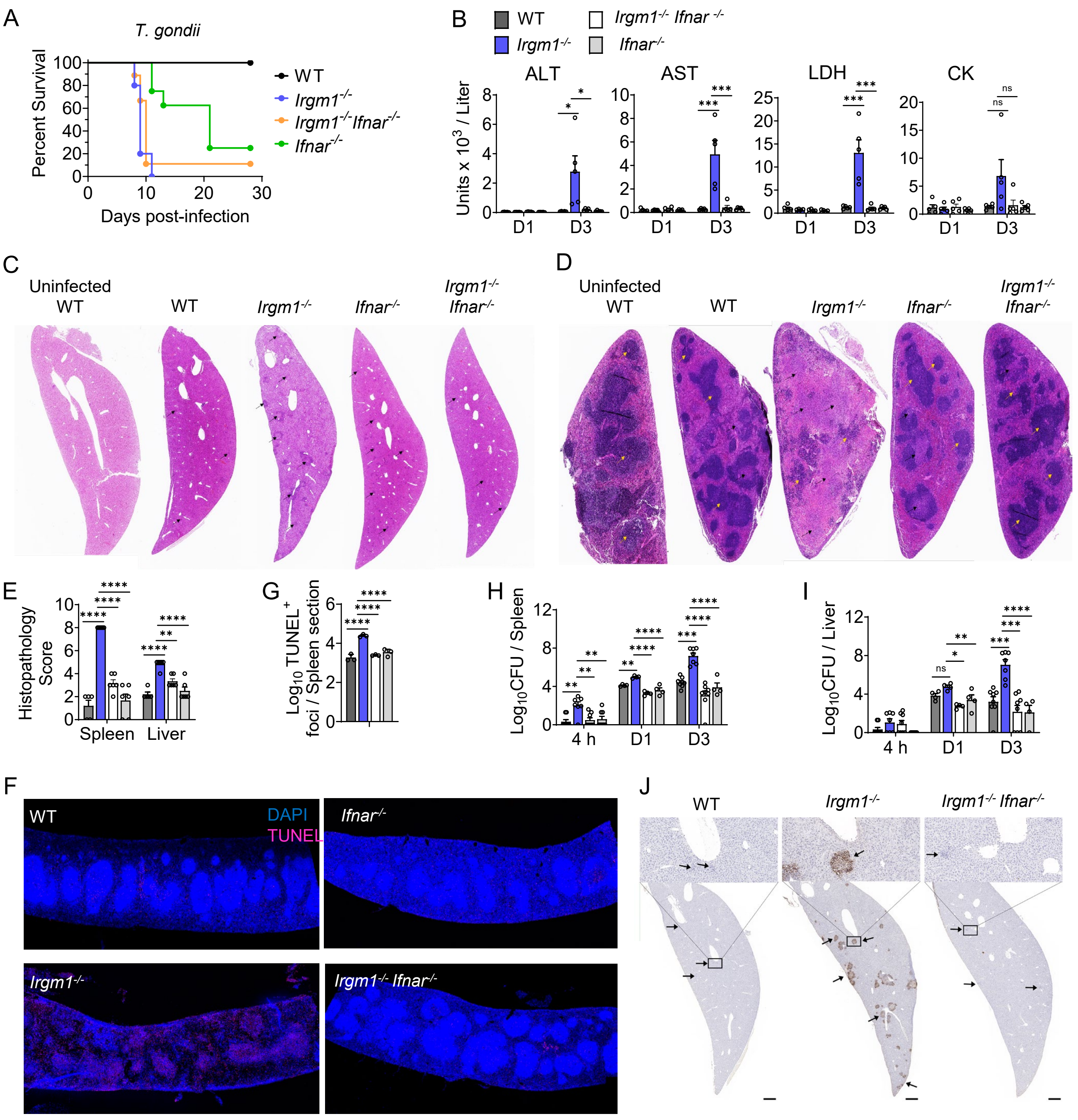

Figure S1. **(A)** WT, *Irgm1*$^{-/-}$, *Ifnar*$^{-/-}$ and *Irgm1*$^{-/-}$*Ifnar*$^{-/-}$ mice were infected with *Toxoplasma gondii* (N=5-9) and survival monitored. **(B)** Alanine aminotransferase (ALT), Aspartate aminotransferase (AST), Lactate Dehydrogenase (LDH) and Creatine Kinase (CK) were measured in serum at day 1 and day 3 post-Listeria infection (N=4-5). Representative H&E staining of **(C)** liver and **(D)** spleen at day 3 post-Listeria infection. Black arrows indicate sites of inflammation and necrosis, yellow arrows indicate splenic white pulp. **(E)** Pathology score of spleen and liver shown in (C) and (D) (N=5-6). **(F)** Spleen at day 3 post-Listeria infection was stained for cell death by TUNEL assay and DAPI for nuclei. **(G)** Quantification of TUNEL^{+} foci (N=3). Listeria CFU in **(H)** spleen and **(I)** liver at 4 hours (4h), day 1 (D1) and day 3 (D3) post-infection (N=4-8). **(J)** Immunohistochemical staining for Listeria showing multiple foci of brown positive staining in *Irgm1*$^{-/-}$ liver. Black arrows indicate areas of inflammation. Scale bar = 500 µm. Data are mean +/− s.e.m. $^{*}P < 0.05$, $^{**}P < 0.01$, $^{***}P < 0.001$ and $^{****}P < 0.0001$ (one-way ANOVA with Tukey's adjustment).

Supplemental Figure 2

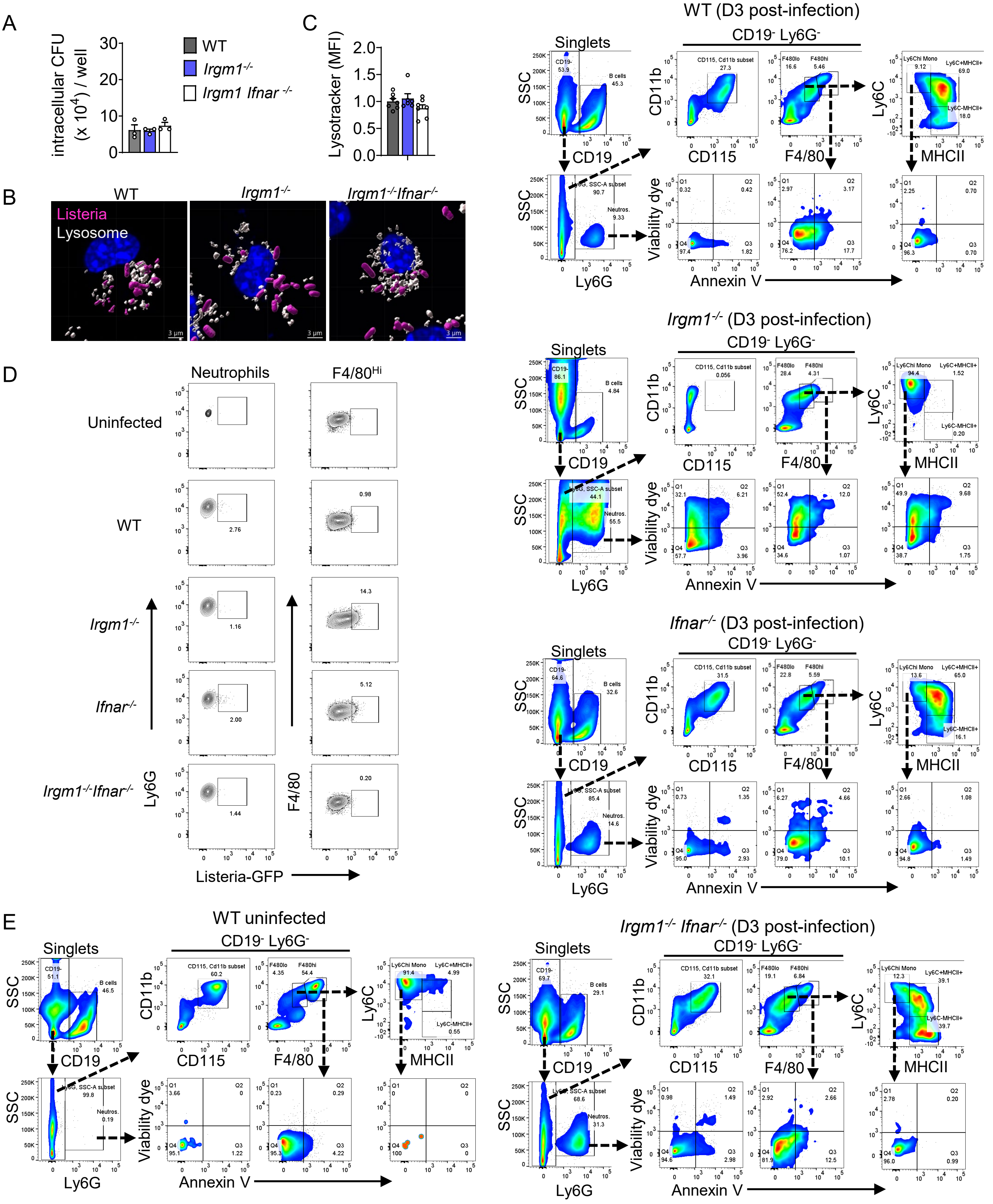

Figure S2. **(A)** Isolated F4/80Hi peritoneal macrophages exposed to Listeria were permeabilized for CFU count after 1h to assess LM internalization. **(B)** Representative 3D surface rendered images of naïve F4/80Hi macrophages that were exposed to Listeria for 6h and stained for Listeria, lysosome (LAMP1) and nuclei (DAPI). Quantification in Figure 1F. **(C)** Lysosomal mass measured by median fluorescence intensity (MFI) of LysoTracker dye in CD11b+F4/80Hi macrophages by Flow cytometry (N=6-7). **(D)** Representative gating strategy for flow cytometry of peritoneal lavage after 4h of infection by GFP-expressing Listeria. Quantification in Figure 1G. **(E)** Representative gating strategy for flow cytometry of peritoneal lavage cells at day 3 post-Listeria infection. Identical gating strategy was used for 4h and day 1 post-infection samples. Quantification in Figure 1I, S3A and S4C.

Supplemental Figure 3

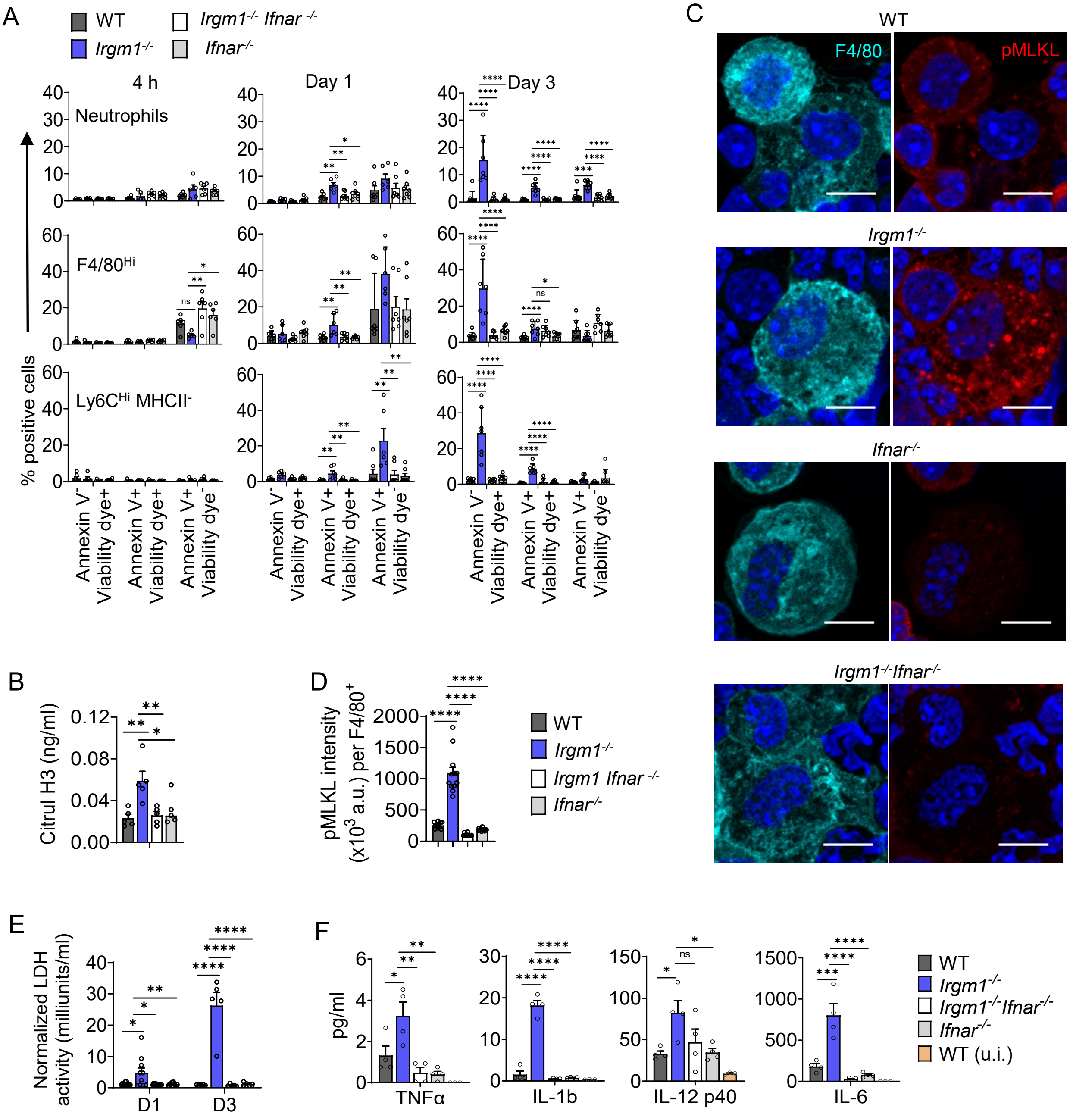

Figure S3. **(A)** Quantification of cell death in peritoneal lavage cells during infection (N=5-8) based on gating strategy shown in Figure S2E. **(B)** Citrullinated histone measurement in cell-free peritoneal lavage at day 3 Listeria-infection (N=5). **(C)** Peritoneal lavage cells at day 1 post-Listeria infection were cytospun and stained for phospho-MLKL (pMLKL, Ser345) and F4/80. **(D)** Quantification of pMLKL intensity in F4/80⁺ cells (N=12 fields of view). Representative of lavage from three animals. **(E)** LDH activity measurement in cell-free peritoneal lavage at day 1 and 3 post-Listeria infection (N=5-10). **(F)** Concentration of cytokines in peritoneal lavage at day 1 post-Listeria (N=4) (u.i. = uninfected). Data are mean +/− s.e.m. #P < 0.08, *P < 0.05, **P < 0.01, ***P < 0.001 and ****P < 0.0001 (one-way ANOVA with Tukey's adjustment).

Supplemental Figure 4

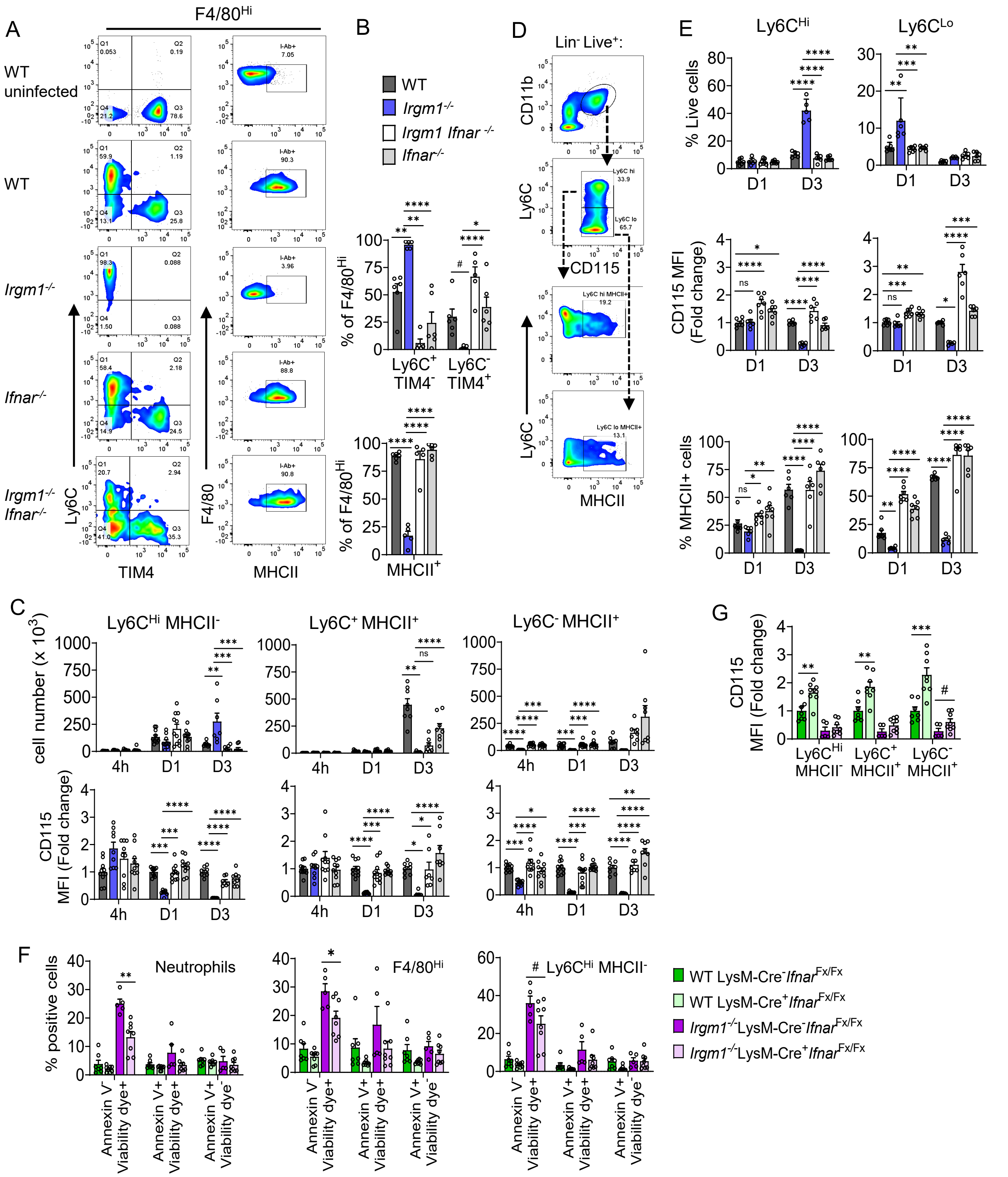

Figure S4. **(A)** Representative gating strategy for flow cytometry of peritoneal F4/80^{Hi} macrophage (parent population gated as Fig. S2E) at day 3 post-Listeria infection. **(B)** Quantification of Ly6C^{+}TIM4^{-}, Ly6C^{-}TIM4^{+} and MHCII^{+} subpopulations within F4/80^{Hi} macrophages (N=5-6). **(C)** Quantification of Ly6C^{Hi} MHCII^{-}, Ly6C^{+} MHCII^{+} and Ly6C^{-} MHCII^{+} populations within the F4/80^{Lo} gate (as shown in Fig. S2E) and their CD115 expression (Median fold change, MFI, compared to WT) (N=7-11). **(D)** Gating strategy for monocytes in blood of WT mice at day 3 post-infection. **(E)** Quantification of Ly6C^{Hi} and Ly6C^{Lo} blood monocytes as well as their MHCII^{+} population and CD115 expression. **(F)** Quantification of cell death in peritoneal lavage myeloid cells of indicated genotypes at day 1 post-Listeria infection (N=5-8). **(G)** CD115 expression in Ly6C^{Hi} MHCII^{-}, Ly6C^{+} MHCII^{+} and Ly6C^{-} MHCII^{+} populations at day 1 post-Listeria infection (N=3-5). Data are mean +/− s.e.m. $^{\#}P \leq 0.1$, $^{*}P < 0.05$, $^{**}P < 0.01$, $^{***}P < 0.001$ and $^{****}P < 0.0001$ (one-way ANOVA with Tukey's adjustment or Student's t-test for 'F' and 'G').
